# Genetic merit of sires for *ad libitum* residual feed intake has no adverse effects on carcass and ham quality traits of restricted-fed heavy pigs

**DOI:** 10.1371/journal.pone.0345035

**Published:** 2026-03-19

**Authors:** Chiara Mondin, Sara Faggion, Valentina Bonfatti, Luigi Gallo, Diana Giannuzzi, Stefano Schiavon, Paolo Carnier

**Affiliations:** 1 Department of Comparative Biomedicine and Food Science, University of Padova, Legnaro (Padova), Italy; 2 Department of Agronomy, Food, Natural Resources, Animals and Environment, University of Padova, Legnaro (Padova), Italy; University of Agriculture Faisalabad, PAKISTAN

## Abstract

This study evaluated the influence of sire genetic merit for residual feed intake (RFI) on carcass and ham quality traits in heavy pigs raised under the restricted feeding conditions typical of Protected Designation of Origin dry-cured ham production. A total of 417 purebred C21 Goland pigs, offspring of 23 sires, were randomly assigned to *ad libitum*, restricted medium-protein, or restricted low-protein dietary treatments. Sire breeding values (EBV) for RFI were estimated using RFI records of *ad libitum*-fed progeny between 96 and 161 kg body weight. Sires were classified into three RFI groups: low EBV (LRFI), medium EBV (MRFI), and high EBV (HRFI). Effects of RFI sire groups were estimated on traits recorded on the restricted-fed progeny. Progeny of LRFI sires exhibited differences in carcass traits, including a significantly higher carcass weight gain (+0.03 kg/day, corresponding to +7.3%; p< 0.01) and increased green ham yield (+1.3%, p < 0.05), compared to those of HRFI sires. LRFI progeny showed no differences in ham fat depth, but exhibited improved subcutaneous fat quality, including higher saturation (increased stearic acid, reduced linoleic acid and polyunsaturated fatty acids) and lower iodine number, as well as firmer subcutaneous fat. No variation in ham weight loss during dry-curing was observed across RFI sire groups. These findings suggest that selection for improved RFI does not significantly compromise carcass and dry-cured ham quality in restricted-fed heavy pigs. Incorporating RFI into selection objectives for sire lines could therefore provide a viable strategy for balancing production efficiency and product quality in heavy pig systems. These findings apply to restricted-fed heavy pigs of the population studied, and potential genotype × feeding regime interactions may limit direct extrapolation to other genetic backgrounds or production systems.

## Introduction

Increasing feed efficiency through selective breeding is a well-established strategy to reduce both the environmental impact and economic costs associated with pig production [1,2]. Among available indicators of feed efficiency, residual feed intake (**RFI**) is widely regarded as a robust metric because it quantifies the difference between an animal’s actual feed intake and the intake predicted from its maintenance and production requirements and, unlike feed conversion ratio, it is largely independent of growth rate and body size [3].

While RFI-based selection has been extensively studied in conventional pig production systems, its implications for specialized production chains remain less explored. In pigs grown from approximately 30 kg to slaughter weight (110–115 kg) under *ad libitum* feeding, selection for lower RFI has generally resulted in leaner carcasses [4,5]. However, findings on meat quality are inconsistent. For example, long-term selection experiments at INRA reported that low-RFI pigs had reduced intramuscular fat content, lower ultimate pH, and higher drip loss compared with high-RFI pigs—differences linked to greater muscle glycogen reserves at slaughter, which promote more extensive post-mortem glycolysis [1,5]. Conversely, no detrimental effects on meat quality or sensory properties were detected in selection experiments at Iowa State University [4,6]. These inconsistencies indicate that the relationship between RFI and meat quality is still unclear, as also evidenced by the inconsistent genetic correlations between RFI and carcass and meat quality traits reported across different populations [5,7]. Notably, these studies were conducted in pigs slaughtered at standard market weights and raised under *ad libitum* feeding conditions.

This leaves a critical knowledge gap regarding the effects of selection for RFI in heavy pigs intended for the production of Protected Designation of Origin (**PDO**) dry-cured hams, where body composition and meat attributes play a critical role in product quality. Subcutaneous fat depth and composition strongly influence the dry-curing process and affect texture, flavor, and overall quality of the final product [8,9]. During dry-curing, fat modulates moisture loss and enzymatic activity, making it a key determinant of processing yield, ham curing weight loss, and sensory profile [10].

Achieving high-quality standards for the raw material typically involves adopting restricted feeding regimes, which are essential for meeting fat deposition requirements. However, this also contributes to significantly higher production costs (20% above the European average) [11] due to extended fattening periods and higher feeding costs, as producing pigs with enhanced fat deposition results in reduced feed efficiency [8,12,13].

Given this context, it is essential to determine whether improvements in feed efficiency, particularly through RFI-based selection, can be achieved without compromising the stringent carcass and fat quality requirements of heavy pig production. Sire EBVs estimate the additive genetic merit transmitted to progeny and integrate information from multiple relatives while accounting for environmental effects, making them a robust predictor of expected progeny performance for traits such as carcass and green ham quality. Because RFI can only be measured in *ad libitum*-fed pigs, and commercial pigs are typically raised under restricted feeding, sire EBVs provide a means to relate sire feed efficiency to progeny performance under practical production conditions. This study addresses the knowledge gap by evaluating how sire classification based on EBVs for RFI (from *ad libitum*-fed progeny) affects carcass and ham quality traits in restricted-fed offspring. Clarifying these effects can inform breeding strategies that optimize production costs, resource use, and product quality, providing evidence-based guidance for sustainable heavy pig production.

## Materials and methods

### Source of data

All animal procedures were reviewed and approved by the institutional animal care committee of the University of Padova, in compliance with European Union legislation governing the use of animals for scientific and educational purposes. Ethical oversight was provided by the “Organismo preposto per il Benessere Animale” (OPBA), under approval number 36/2018. Written informed consent was obtained from the animal owner to authorize participation in the study.

Findings on *in vivo* traits of restricted-fed pigs from this project have been published in a previous study [14].

The experimental dataset, described in detail by Mondin et al. [14], included 417 purebred C21 Goland pigs (Gorzagri, Fonzaso, Italy) from four consecutive rearing batches of 97–111 animals each. Pigs were progeny of 23 different sires, with an average of 18 ± 9 offspring per sire. Animals were transferred from a commercial farm to the experimental station of the University of Padova (Legnaro, Italy) at ~94 kg body weight and 148 days of age and were randomly assigned to three feeding treatments, according to a split-plot design: 1) *ad libitum* high-protein diet (n = 205; 4 pens per batch); 2) restricted medium-protein diet (**MP**; n = 104; 2 pens per batch); 3) restricted low-protein diet (**LP**; n = 108; 2 pens per batch). Animals were randomly assigned to feeding treatments while ensuring comparable initial weight and balanced sexes. Each sire had progeny in all dietary groups. Batch was included as a blocking factor. Feeding treatment was applied at the whole-plot level, with the pen as the whole-plot unit, since all pigs in the same pen received the same treatment. Individual pigs were measured within pens, representing the subplot unit. Pigs were housed in pens of 12–15 animals (5.8 × 3.8 m; 1.57 m²/pig), with individual feed intake recorded via single-space electronic feeders (Compident Pig–MLP, Schauer Agrotronic, Prambachkirchen, Austria). Diet composition and nutrient content are provided in the Supplementary Material (S1 Table).

The *ad libitum* group was designed to provide data to estimate sire breeding values (**EBV**) for RFI, which were subsequently used to classify sires according to their genetic merit for RFI. Data collected from restricted-fed pigs (MP and LP groups) were used to assess the influence of RFI sire classification on carcass and ham quality traits. The MP treatment reflected standard practices in Italian heavy pig production, targeting slaughter at 9 months of age and 170 kg body weight. The LP group was included as an attempt at reducing feeding costs, nitrogen emissions, and to promote animal fat tissue deposition. It shared the same slaughter weight target as the MP group, but was expected to reach it at an older age, due to limited dietary protein and reduced levels of ileal digestible lysine. Including different treatments allowed the study of dietary differences in the analysis of growth and carcass traits, and ham quality [14–16] without increasing experimental complexity, while maintaining sufficient replication within each treatment.

After a 6-day post-arrival adaptation, the duration of the experimental period ranged from 67 days (*ad libitum* group) to 135 days (LP), after which the animals were slaughtered.

### Post-mortem data collection

All animals were slaughtered at the same commercial facility (OPAS, Carpi, Italy) across 12 slaughter dates, according to batch and dietary treatment. Carcass processing followed standard commercial procedures as described by Malgwi et al. [15]. Feed distribution was suspended on the day preceding slaughter. Pigs were weighed and transported to the slaughterhouse. After 2 h of resting at the slaughterhouse, animals were stunned using a high concentration of carbon dioxide and slaughtered by exsanguination through jugular vein severance, in accordance with standard commercial slaughterhouse procedures. Individual hot carcass weight was recorded and used to calculate carcass yield. Carcass lean meat percentage was assessed using the Fat-O-Meater (Carometec, Soeborg, Denmark) following the methodology outlined in European Directive 2014/38/UE. Hot carcasses were partitioned into commercial lean cuts (loin with ribs, shoulder, and ham) and fat cuts (lard and belly), which were individually weighed. The weight of each cut was expressed as a percentage of the total carcass weight. The left ham from each carcass was used for green ham quality assessment. Muscle pH was measured in triplicate, 30 minutes post-mortem, on the *semimembranosus* muscle using a portable pH meter (HI-8314, Delta Ohm, Padova, Italy). Ham quality traits were assessed at two stages: on green hams and after completion of the dry-curing process.

### Green ham quality assessment

After a 24-hour cooling period (1–2°C), the green ham was trimmed to obtain the characteristic round shape and weighed (trimmed ham weight). pH measurements were taken on trimmed hams using the same method as for post-mortem pH. Subcutaneous fat depth measurements were collected in proximity of the *biceps femoris* and *semimembranosus* muscles using a gauge and a portable ultrasound system (Aloka SSD 500 equipped with UST-5512 7.5 MHz linear transducer probe, Hitachi Medical Systems S.p.A., Milan, Italy), respectively. Using a linear grading system, a trained expert evaluated all trimmed hams for round shape (0 = low to 4 = high), veining (0 = absent to 4 = evident), visible marbling (0 = low to 4 = high), fat cover depth (−4 = low to 4 = high), and lean muscle color (−4 = pale to 4 = dark). Carcass and green ham quality trait records were available for 414 out of 416 pigs; the two pigs without records belonged to the *ad libitum*-fed group.

For 361 pigs, of which 182 restricted-fed, visible-near infrared predictions of the percentage of linoleic acid (C18:2n-6), stearic acid (C18:0), polyunsaturated fatty acids (PUFA), monounsaturated fatty acids (MUFA) to PUFA ratio, and iodine number in subcutaneous fat were obtained for all hams as detailed in Bonfatti et al. [17]. Infrared prediction model validation and accuracy are also described in Bonfatti et al. [17]. The values of R^2^ obtained in validation for C18:2n-6, C18:0, PUFA, MUFA to PUFA ratio, and iodine number were 0.77, 0.63, 0.76, 0.73, and 0.72, respectively.

### Ham weight assessment

Hams of pigs with available carcass and green ham quality traits records (n = 414) were transferred to a ham factory (“Attilio Fontana Prosciutti”, Montagnana, Italy), and processed according to Prosciutto Veneto Berico-Euganeo PDO specification [18], whose requirements for the raw material and processing phases are comparable to those of Parma ham. Processing steps were described in detail in Toscano et al. [16].

At the ham factory, hams were weighed at arrival and initially at 1-week intervals (7 ± 1 days, first salting; 13 ± 1 days; 20 ± 1 days; 31 ± 3 days, end of second salting). Later, ham weight was recorded at 144 ± 11, 151 ± 10, and 188 ± 14 days (resting), 416 ± 22 days (14 months, potential ending point of the dry-curing process) and at completion of the dry-curing process (604 ± 21 days). This resulted in a total of 10 weight measurements per ham. Ham cumulative weight loss (**CWL**) was computed as a percentage of the initial trimmed ham weight.

### Modelling of the dynamics of ham weight loss during dry-curing

Following the approach reported by Bonfatti et al. [19], the non-linear Chapman-Richards model [20] was fitted, for each ham, to all the available CWL records (n = 10, including the CWL at arrival which was equal to 0), and used to estimate parameters of the individual curves describing the within-pig variation of CWL during dry-curing. The model was the following:


$$y_{t}=A\times [1-exp(-kt){]}^{b}$$ (1)

where $y_{t}$ is the expected CWL (%) of an individual ham at time t (day of dry-curing); A (%) is the upper asymptotic CWL achievable for time → ∞; k (1/day) is the coefficient of loss affecting the steepness of the resulting curve, indicating how rapidly CWL approaches A; b is the curve shape parameter, controlling the curvature of the trajectory (i.e., how sharply or gradually weight loss decelerates over time). When b > 1, the curve has a sigmoidal shape, characterized by a distinct inflection point and a gradual acceleration followed by deceleration. When b < 1, the curve describes a continuously decelerating process where the rate of weight loss is the highest at the beginning, decreases markedly in the initial days, and keeps decreasing later, but at a progressively lower rate.

A nonlinear mixed-effects formulation was not adopted because the simultaneous inclusion of multiple design factors (e.g., batch, pen, sex, sire class, treatment) at the nonlinear fitting stage would have substantially increased model complexity and reduced parameter interpretability. Estimating individual ham-specific curves allowed us to derive biologically meaningful descriptors of CWL dynamics that were subsequently used as response variables in further statistical analyses.

The model parameters were estimated using the function nlsLM in the minpack.lm package of the R software v.4.2.3 [21]. The symbolic first derivative of the Chapman-Richards model was obtained using the nlsDeriv function from the R package nlsr. Then, the estimates of the parameters of each individual CWL curve were substituted into the derivative function, generating the corresponding curves of instantaneous rate of absolute weight loss (%/day). The instantaneous rate of absolute weight loss, obtained from the model’s first derivative, was interpreted as a relative indicator of drying dynamics rather than a direct measure of evaporation rate, since actual moisture transfer is influenced by multiple physicochemical processes.

### Dry-cured ham quality assessment

At the end of dry-curing, each ham was evaluated at the processing facility by a trained expert using a linear scoring system. Before deboning, the following traits were scored: round shape (1 = low to 3 = high), subcutaneous fat depth (1 = low to 5 = high), subcutaneous fat firmness (1 = soft to 3 = firm), and subcutaneous fat color (1 = yellow to 3 = white). After deboning, additional quality traits were assessed, including presence of fat vein between the *semimembranosus* and *semitendinosus* muscles extending from subcutaneous fat (1 = absent to 3 = pronounced), marbling (1 = low to 5 = high), muscle firmness assessed by manual pressure (1 = soft to 5 = firm), lean meat color (1 = pale to 5 = dark), muscle separation (1 = absent to 3 = severe), and firmness assessed by insertion of a horse fibula at multiple muscle sites (1 = low to 5 = high). Although this method is primarily used for olfactory assessment of curing progress, it also provides indications of muscle resistance to penetration and texture, which are influenced by the proteolytic and enzymatic activity essential to the development of dry-cured ham flavor and structure [11, 22].

### Sire classification based on estimated breeding value for residual feed intake

As the estimation of RFI requires unrestricted feed access, calculations were limited to pigs of the *ad libitum* group for which RFI (kg/day) was computed as described by Mondin et al. [14].

Breeding values for RFI were estimated for the 23 sires using a linear mixed animal model fitted to the RFI data of the *ad libitum*-fed progeny (n = 205; from 3 to 25 pigs per sire). The model, implemented in BLUPF90 [23], included the random effect of the batch (4 levels) and the random additive genetic effect of the animal. Additive genetic relationships were estimated from a pedigree file comprising 1909 animals across 22 generations, including 205 individuals with RFI records and 1702 ancestors.

To reflect selection practices and evaluate progeny responses across contrasting levels of genetic merit for RFI, including potential non-linear effects, sires were classified into three RFI classes based on their estimated breeding value (**EBV**): low-RFI (**LRFI**, 25% lowest EBV, n = 6), medium-RFI (**MRFI**, 50% intermediate EBV, n = 11), and high-RFI (**HRFI**, 25% highest EBV, n = 6). Although the number of progeny per sire ranged from 3 to 25, most sires had a sufficient number of offspring to produce reliable EBVs. Furthermore, average EBV reliability was similar across sire classes, indicating comparable accuracy among the three groups. Descriptive statistics of sire EBVs by RFI class are reported in Mondin et al. [14].

### Effects of sire classification for RFI on carcass and green ham quality traits of the restricted-fed progeny

The effects of sire classification on carcass and green ham quality traits of the restricted-fed progeny were estimated using data from both the MP and LP dietary groups jointly. On average, each sire had 9 ± 5 restricted-fed offspring (range: 3–23), with all sire classes represented across both feeding treatments: in the MP group there were 30, 45, and 29 pigs from LRFI, MRFI, and HRFI sires, respectively, whereas in the LP group pigs were 40, 36, and 32, respectively.

Data on carcass and green ham traits were analyzed using the following mixed linear model:


$${\text{y}}_{ijklmn}=\;\mu \;+{\text{sex}}_{i}+{\text{sire class}}_{j}+{\text{batch}}_{k}+{\text{treatment}}_{l}+\text{ pen }(\text{batch}\times \text{treatment}{)}_{m:kl}{+\text{ e}}_{ijklmn}$$ (2)

where y was the observed trait, µ was the overall intercept, sex was the fixed effect of sex (*i*: 1 = barrow, 2 = gilt), sire class was the fixed effect of the sire class for RFI (*j*: 1 = LRFI, 2 = MRFI, 3 = HRFI), batch was the random effect of the batch (*k*: 1, …, 4), treatment was the fixed effect of the treatment group (*l*: 1 = MP, 2 = LP), and pen (batch × treatment) was the random effect of the pen nested within the batch × treatment interaction. Differences between MP and LP groups were tested using the pen (batch × treatment) interaction as the error term, while sex and sire class were tested against the residual variance. As the primary objective was to evaluate the effect of sire class on progeny traits, treatment was included only to adjust for potential dietary differences and was not reported. Similarly, sex effects were not reported. All statistical analyses were conducted in R v.4.2.3 [21] using the *lmerTest* and *lsmeans* packages.

Differences between the mean of LRFI and that of HRFI or MRFI group were tested in F-tests, rejecting the null hypothesis of equal means when p < 0.05.

The same model fitted to carcass and green ham data was also used to investigate the effects of sire classification on parameters of the individual CWL curves. The least square means of the sire class effects on the parameters of individual curves were then used as parameters of the average curve of each sire class. Differences between CWL curves of RFI groups were tested using F-tests on the Chapman-Richards model parameters *A*, *k*, and *b*. Curves, as well as their first derivatives, were considered significantly different when at least one of these parameters differed across sire classes (p < 0.05).

### Effects of sire classification for RFI on dry-cured ham quality attributes of the restricted-fed progeny

To analyze dry-cured ham quality traits, proportional-odds cumulative logistic models were applied using the *clmm* function of the *ordinal* package in R v.4.2.3 [21]. These models are appropriate for ordinal outcomes and estimate the cumulative probability of a response falling within or below a given category based on values of a latent continuous variable which depend on the model predictors. The model estimated the log cumulative odds of assignment to a category as a function of fixed effects (sex, feeding treatment, and sire classification for RFI) and random effects (batch). Being nominal variables, these effects were included in the model as a set of dummy variables. Odds ratios (**OR**) were calculated by exponentiating the base of natural logarithms to the estimated value of the regression coefficients.

This approach was used as class merging was necessary due to sparse data in some categories, which led to a reduction in the number of usable response levels. In particular, categories with frequencies below 5% were merged with adjacent categories, resulting in 3 or 4 ordered categories per trait: 4 categories for subcutaneous fat depth (from 2 to 5), marbling (from 1 to 4), lean muscle color (from 1 to 4), and firmness assessed by fibula insertion (from 2 to 5), and 3 categories for muscle firmness assessed by manual pressure (from 3 to 5).

## Results

### Effects of sire classification for RFI on carcass and green ham quality traits of restricted-fed progeny

The least squares means for the effects of RFI sire classification on carcass traits of the restricted-fed progeny are presented in Table 1. Progeny of LRFI sires showed a significantly higher carcass weight gain than progeny of HRFI sires (+7.3%; p< 0.01). Carcass yield was also slightly higher in the LRFI group (+0.6%) than in the HRFI group (p = 0.052). No significant differences across sire classes were detected for lean meat content or backfat depth, although LRFI pigs tended to exhibit a lower lean meat percentage compared with HRFI (p = 0.094).

**Table 1:** Effects of sire classification for residual feed intake (RFI) on carcass traits of restricted-fed pigs.

Trait	Class of sire for RFI^a^			LRFI vs MRFI (p-value)	LRFI vs HRFI (p-value)
	HRFI (n = 61)	MRFI (n = 81)	LRFI (n = 70)		
Carcass weight gain (kg/day)	0.372 ± 0.024	0.386 ± 0.024	0.399 ± 0.024	0.136	0.005
Carcass yield (%)	80.90 ± 0.25	81.00 ± 0.23	81.40 ± 0.24	0.135	0.052
Lean meat content (%)	51.60 ± 0.55	51.00 ± 0.52	50.80 ± 0.53	0.562	0.094
Backfat depth (mm)	38.80 ± 0.82	38.50 ± 0.75	39.30 ± 0.77	0.346	0.582
Commercial cut yield, % carcass weight					
Total cuts	71.90 ± 0.42	72.00 ± 0.42	72.20 ± 0.42	0.246	0.200
Fat cuts	19.10 ± 0.41	19.50 ± 0.40	19.60 ± 0.41	0.865	0.065
Belly	12.00 ± 0.20	12.20 ± 0.19	12.20 ± 0.19	0.689	0.253
Lard	7.11 ± 0.37	7.31 ± 0.37	7.38 ± 0.37	0.621	0.081
Lean cuts	52.80 ± 0.32	52.40 ± 0.31	52.60 ± 0.31	0.420	0.516
Loin with ribs	15.20 ± 0.13	15.20 ± 0.12	15.10 ± 0.12	0.535	0.258
Shoulder	13.90 ± 0.19	13.50 ± 0.18	13.60 ± 0.19	0.484	0.008
Green ham	23.70 ± 0.10	23.80 ± 0.09	24.00 ± 0.09	0.086	0.029
Trimmed ham	19.60 ± 0.13	19.50 ± 0.13	19.70 ± 0.13	0.080	0.467
Average pH					
at 30 min *post-mortem*	6.15 ± 0.08	6.12 ± 0.08	6.16 ± 0.08	0.363	0.876
at 24 h *post-mortem*	5.74 ± 0.06	5.71 ± 0.06	5.72 ± 0.06	0.650	0.649

Least squares means (± SE) for the effects of sire class on carcass traits of the restricted-fed progeny.

^a^HRFI: high-RFI sire class; MRFI: medium-RFI sire class; LRFI: low-RFI sire class.

No significant differences were observed for total, fat, or lean cuts. However, when compared with HRFI progeny, LRFI pigs exhibited significantly lower shoulder yield (−2.2%; p < 0.01), higher ham yield (+1.3%; p < 0.05), and a tendency towards a higher fat cut yield (p = 0.065) that can be ascribed to a slightly higher lard yield (p = 0.081).

After trimming, ham yield of LRFI offspring was not significantly different from that of HRFI progeny. No significant differences were observed among groups for average pH measured at either 30 minutes or 24 hours post-mortem.

The least squares means for the effects of sire classification on green ham quality traits of restricted-fed offspring are reported in Table 2. No significant differences were observed among sire RFI sire classes for subcutaneous fat depth measured near the *biceps femoris* or *semimembranosus* muscles, nor for green ham scores.

**Table 2:** Effects of sire classification for residual feed intake (RFI) on green ham traits of restricted-fed pigs.

Trait	Class of sire for RFI^a^			LRFI vs MRFI (p-value)	LRFI vs HRFI (p-value)
	HRFI (n = 61)	MRFI (n = 81)	LRFI (n = 70)		
Subcutaneous fat depth (mm) in the proximity of					
*biceps femoris*	28.10 ± 1.40	27.40 ± 1.35	28.10 ± 1.37	0.451	0.953
*semimembranosus*	6.86 ± 0.22	6.67 ± 0.21	6.67 ± 0.22	0.980	0.253
Green ham scores					
Round shape	1.24 ± 0.14	1.47 ± 0.13	1.33 ± 0.13	0.382	0.622
Veining	1.17 ± 0.13	1.34 ± 0.12	1.31 ± 0.12	0.833	0.364
Visible marbling	0.87 ± 0.10	0.83 ± 0.09	0.69 ± 0.09	0.256	0.190
Fat cover thickness	0.03 ± 0.34	0.15 ± 0.33	0.34 ± 0.33	0.386	0.173
Lean muscle color	−1.06 ± 0.47	−1.05 ± 0.46	−0.86 ± 0.47	0.371	0.368

Least squares means (± SE) for the effects of sire class on green ham traits of the restricted-fed progeny.

^a^HRFI: high-RFI class; MRFI: medium-RFI class; LRFI: low-RFI class.

Sire classification for RFI significantly influenced the subcutaneous fat composition of hams from restricted-fed pigs (Table 3). Compared with pigs sired by HRFI boars, those from LRFI sires exhibited a 3.9% lower iodine number (p < 0.001), indicating a more saturated fat profile. The proportion of stearic acid (C18:0) in LRFI pigs was 4.8% higher than in HRFI pigs (p = 0.008).

**Table 3:** Effects of sire classification for residual feed intake (RFI) on subcutaneous ham fat quality of restricted-fed pigs.

Trait^a^	Class of sire for RFI^b^			LRFI vs MRFI (p-value)	LRFI vs HRFI (p-value)
	HRFI (n = 47)	MRFI (n = 69)	LRFI (n = 66)		
Iodine number	66.50 ± 2.30	65.40 ± 2.29	63.90 ± 2.29	0.014	< 0.001
C18:0 (g/100 g fat)	12.40 ± 1.64	12.70 ± 1.63	13.00 ± 1.63	0.082	0.008
C18:2n-6 (g/100 g fat)	15.70 ± 0.43	15.10 ± 0.41	14.40 ± 0.41	0.026	< 0.001
PUFA (g/100 g fat)	18.50 ± 0.50	17.90 ± 0.48	17.00 ± 0.48	0.026	< 0.001
MUFA/PUFA	2.49 ± 0.17	2.62 ± 0.17	2.81 ± 0.17	0.043	0.002

Least squares means (± SE) for the effects of sire class on subcutaneous fat quality of the restricted-fed progeny.

^a^C18:0: stearic acid; C18:2n-6: linoleic acid; PUFA: polyunsaturated fatty acids; MUFA: monounsaturated fatty acids.

^b^HRFI: high-RFI class; MRFI: medium-RFI class; LRFI: low-RFI class.

Linoleic acid (C18:2n-6) content in LRFI pigs was 8.3% lower than that in HRFI pigs (p < 0.001), while the overall PUFA content was 8.1% lower (p < 0.001). Furthermore, LRFI pigs showed a 12.9% higher MUFA/PUFA ratio than HRFI pigs (p = 0.002), indicating a shift towards a more monounsaturated fatty acid profile.

Compared with MRFI pigs, the LRFI group had a 2.3% lower iodine number (p = 0.014), 6.4% lower C18:2n-6 content (p = 0.026), 5% lower PUFA content (p = 0.026), and a 7.3% higher MUFA/PUFA ratio (p = 0.043). The stearic acid content in LRFI pigs tended to be higher than that of MRFI pigs, but the difference was not statistically significant (p = 0.082).

### Effects of sire classification for RFI on dry-cured ham quality traits of restricted-fed progeny

**Fig 1:**
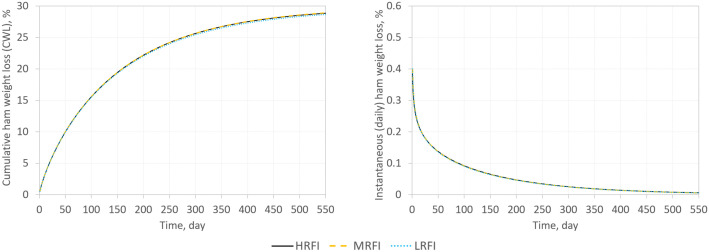
Dynamics of cumulative ham weight loss and instantaneous rate of absolute weight loss during dry-curing in the restricted-fed progeny of RFI sire classes. Genetic merit of sires for RFI was used to classify sires into low-, medium-, and high-RFI (LRFI, MRFI, and HRFI, respectively). Parameters of the curves are not different across sire classes (p > 0.05).

Fig 1 illustrates the dynamics of CWL and instantaneous rate of absolute weight loss during the dry-curing process of the restricted-fed progeny of the RFI sire classes. The dynamics of CWL exhibited similar patterns across sire classes, with a fast increase in the early stages of curing and a gradual deceleration over time. The instantaneous rate of absolute weight loss peaked at the beginning of the curing period and decreased as the process progressed.

No significant differences were observed among RFI sire classes in the parameters of the CWL curves (p > 0.05; S2 Table), indicating that sire genetic merit for RFI did not affect the dynamics of ham dehydration in restricted-fed progeny during dry-curing.

**Fig 2:**
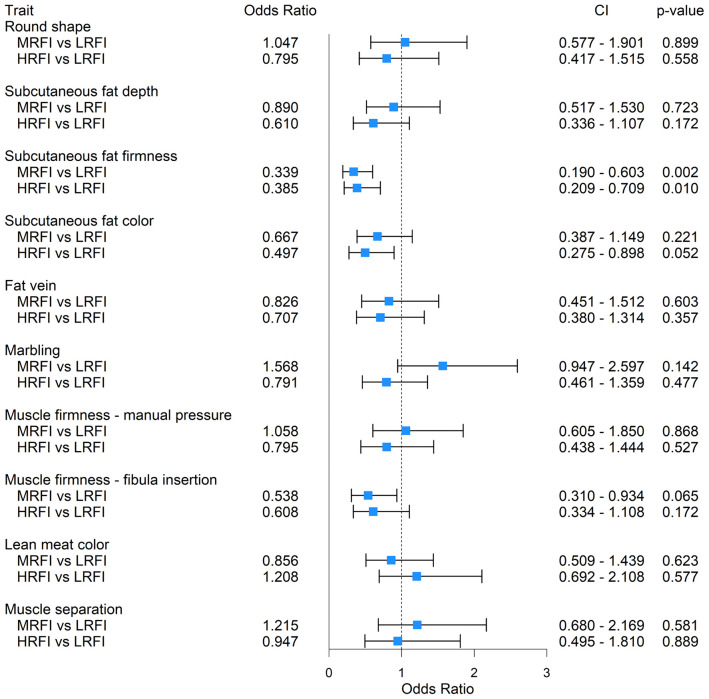
Odds ratios for the effect of sire classes for residual feed intake on dry-cured ham quality attributes of restricted-fed pigs. Genetic merit of sires for RFI was used to classify sires into low-, medium-, and high-RFI. The dashed line corresponds to an odds ratio equal to 1.00.

Odds ratios, confidence intervals, and *p*-values for the effects of sire classification on dry-cured ham quality of the restricted-fed progeny are presented in Fig 2.

Subcutaneous fat firmness was significantly lower in the progeny of MRFI and HRFI sires than in the progeny of LRFI sires. Specifically, the odds ratio for fat firmness was 66.1% lower in the MRFI group (OR = 0.339, p = 0.002) and 61.5% lower in HRFI group (OR = 0.385, p= 0.010) than in LRFI pigs, indicating an increase in the probability of obtaining low scores (i.e., soft subcutaneous fat) in hams from the MRFI and HRFI groups. A tendency toward a yellower subcutaneous fat color was also observed in the HRFI group compared with the LRFI group (OR = 0.497, p = 0.052). No significant differences among sire classes were detected for other ham traits, including round shape, subcutaneous fat depth, fat vein presence, marbling, muscle firmness (assessed by manual pressure or fibula insertion), lean meat color, or muscle separation (p > 0.05 for all comparisons).

## Discussion

This study investigated the effect of sire classification based on EBV for RFI on carcass and ham quality traits of the restricted-fed progeny. While the effects of sire RFI classification on feed efficiency, growth performance, and body composition of the restricted-fed progeny have been previously reported [14], the current analysis extends these findings to traits directly relevant to dry-cured ham production.

RFI was assessed in the weight interval from 96 kg to 161 kg body weight, representing a later stage of development compared to most existing studies, which typically evaluate RFI up to 100–115 kg body weight [4,5,7]. As such, the carcass and ham traits investigated in this study reflect feed efficiency differences during a physiologically distinct growth phase. For this reason, comparisons with results from other studies should be made with caution.

To our knowledge, this is the first study exploring the relationship between RFI and ham quality traits. Previous research on the relation between feed efficiency and meat quality has been predominantly focused on the *longissimus* muscle, with inconsistent findings regarding its composition, sensory attributes, and water-holding capacity [5,6,24,25]. In contrast, the present study evaluates quality attributes of relevance for PDO dry-cured ham production. Despite the limited sample size, which is an inherent constraint in heavy pig testing due to its operational and economic demands, this work provides novel insights into the potential compatibility between genetic improvement for RFI and high-quality product standards.

### Effects of sire RFI classes on carcass traits of restricted-fed pigs

The limited sample size inherent to heavy pig experimentation may have reduced statistical power, potentially preventing some differences from reaching conventional significance thresholds. Nevertheless, beyond formal hypothesis testing, the estimated means indicated coherent trends across traits, providing biologically meaningful information on the direction and magnitude, or lack thereof, of potential effects.

In addition to consuming less feed [14], progeny of LRFI sires exhibited greater carcass weight gain and higher ham yield compared to those of HRFI sires. Interestingly, results indicate that carcass adiposity did not differ between sire classes, and the absence of statistical significance along with the very small differences between classes can be considered a favorable outcome in this production context. Progeny of LRFI sires also tended to show improved carcass yield, reduced lean meat content, and a higher proportion of fat cuts, even though differences were not significant.

Carcass attributes observed in LRFI progeny may enhance farmers’ profitability, as the commercial value of heavy pigs is closely linked to slaughter weight, carcass yield, and body composition. In the Italian market, increased carcass weight is advantageous for dry-cured ham production, as it is generally associated with reduced curing loss [26]. Moreover, only carcasses graded as EUROP “U”, “R”, or “O” are eligible for PDO dry-cured ham production [27], a classification that favors intermediate lean-to-fat ratios. The observed increase in ham weight in LRFI offspring is particularly beneficial, as the ham represents the most valuable cut, contributing over 25% of total carcass value [28], and a greater ham weight is associated with improved sensory quality [10] and lower deboning losses [26].

Our findings diverge from previous studies on both *ad libitum*- and restricted-fed pigs [29–31] where more feed-efficient animals had leaner carcasses and lower fat deposition. Such discrepancies likely reflect differences in slaughter weight, breed, and physiological development stage. Our results also contrast with the well-established negative relationship between feed conversion ratio and lean meat content in pig breeds [32], highlighting the distinct biological meaning and practical implications of alternative feed efficiency indicators.

As suggested by Mondin et al. [14], under restricted feeding, pigs with lower maintenance energy requirements or greater efficiency in nutrient utilization can achieve either comparable growth with reduced intake or accelerated growth at the same intake level. Animals with superior feed efficiency or reduced maintenance energy requirements, such as those from LRFI sires, can allocate more dietary energy toward tissue deposition. As protein deposition rates typically peak between 60 and 80 kg body weight and inherently decline in the late finishing phase [33], this shift in energy partitioning tends to favor lipid accumulation in heavier pigs. Consistent with this, Mondin et al. [14] reported a tendency for increased fat deposition in LRFI progeny at older ages.

### Effects of sire RFI classes on green ham quality

Green ham quality traits were largely unaffected by sire RFI class. Subcutaneous fat depth in both the *biceps femoris* and *semimembranosus* regions and visual evaluations of fat cover depth at the ham factory showed no variation across sire classes, indicating that selection for improved RFI does not impair the physical quality of green hams. This is particularly relevant for PDO dry-cured ham production, where adequate fat cover and distribution are crucial to ensure optimal curing dynamics and sensory characteristics of end products [10]. These findings align with the observed carcass composition and suggest that the potential for leaner carcasses of LRFI pigs, commonly expressed under *ad libitum* feeding and at lighter target body weight [1,5], is mitigated under the heavy-weight, restricted-feeding conditions typical of PDO farming systems. Restricted feeding likely redirects the energy savings from improved feed efficiency toward fat deposition, ensuring adequate fat levels required for ham processing.

Supporting this hypothesis, Mondin et al. [1 4] reported that restricted-fed progeny of sires with divergent genetic merit for RFI did not differ in body weight gain composition, suggesting a compensatory mechanism that preserves fat deposition in more efficient animals when feed intake is restricted. These findings suggest that RFI can be effectively integrated into selective breeding programs for heavy pigs, supporting both economic sustainability and compliance with the strict quality standards of PDO production systems.

### Effects of sire RFI classes on fatty acid composition

Progeny of LRFI sires exhibited a more favorable fatty acid composition than progeny of HRFI sires. Fatty acid composition, particularly the balance between saturated fatty acids (SFA) and PUFA, and the associated iodine number are critical determinants of fat quality in dry-cured hams [8]. An excess of PUFA in fat, particularly linoleic acid, can lead to undesirable features such as poor fat firmness, increased oiliness, compromised tissue cohesion, and increased susceptibility to oxidative breakdown, which in turn results in rancidity, off-flavor, and alterations in color and firmness of the final product [8,10,34]. This poses a particular challenge for products with extended curing periods, like PDO dry-cured hams, which, for this reason, must comply with specific cut-offs for iodine number and linoleic acid content [27].

In pigs, fatty acids originate either from endogenous *de novo* lipogenesis (primarily MUFA and SFA) or are absorbed unchanged from the diet (typically PUFA) [35]. Hence, the balance between these two sources largely determines overall fat composition. The more saturated fat profile in LRFI pigs could be related to two potential mechanisms: i) LRFI pigs may have extra energy available for *de novo* fatty acid synthesis, thereby favoring SFA accumulation; ii) restricted-fed LRFI pigs consumed approximately 3% less feed on average than MRFI and HRFI counterparts [1 4], potentially leading to reduced dietary PUFA intake and, consequently, a lower proportion of unsaturated fatty acids in the adipose tissue. This shift in fatty acid composition may have positive implications for the quality of dry-cured products, where a higher SFA content is considered desirable. While the proposed mechanisms explaining differences in fatty acid composition are speculative, they are consistent with known pathways of lipid metabolism in pigs; future studies including detailed metabolic or dietary measurements would help confirm these interpretations.

### Effects of sire RFI classes on ham weight loss and dry-cured ham quality attributes of restricted-fed pigs

Sire RFI classification did not affect the CWL dynamics, indicating that an enhanced sire genetic merit for RFI is not expected to alter the basic drying behavior of hams during processing, which is critical for achieving optimal dehydration without adverse effects on the yield of the marketable product, its salt concentration, and the development of the typical flavour and texture [10].

Pigs sired by LRFI boars were less likely to exhibit undesirable features such as softness and yellowish color of subcutaneous fat compared with progeny of MRFI and HRFI sires. These traits are crucial for the PDO dry-cured ham sector. Firm subcutaneous fat is essential for optimal protection of the underlying muscle during curing, minimizing oxidative deterioration and the formation of undesirable volatile and non-volatile compounds. Fat firmness and color are primarily determined by the fatty acid composition of fat, particularly the proportion of PUFA, which, if high, increases fat softness and discoloration due to oxidative instability [10,36,37].

No significant effects of sire classification were observed for other ham traits, including round shape, marbling, fat veining, muscle firmness, lean meat color, and muscle separation. From the perspective of the PDO market, this lack of variation across sire classes is also favorable. An increased ham roundness is often associated with leaner pigs, leading to excessive curing losses and diminished sensory quality in the final dry-cured product, also due to lower salt diffusion into the ham [38]. While fat veining is considered a defect that negatively impacts consumer acceptance, marbling plays a crucial role in enhancing aroma, juiciness, and flavor intensity during the curing process [26], despite also influencing consumer preference. Preserving marbling within an optimal and uniform range is crucial to achieving consistent sensory quality and maximizing product appeal. Similarly, lean meat color and firmness are critical for both visual appeal and slicing performance. Deviations in color may indicate oxidative instability or abnormal post-mortem muscle metabolism, which can negatively impact both appearance and flavor [39,40]. Muscle firmness, primarily influenced by the extent of proteolysis and water-holding capacity, directly affects slicing precision, which is an essential quality parameter for thin-sliced, premium dry-cured products [41,42]. Muscle separation, referring to the cohesion and integrity of muscle bundles and their attachment to surrounding tissues, is also crucial. Loss of this structural integrity can lead to defects such as cracking, detachment, or deformation during prolonged curing and deboning processes, thereby compromising the commercial value of the final product and process efficiency [9].

Excessive deviations in any of these characteristics could result in downgrading or rejection of hams under PDO standards. The absence of undesirable shifts in these traits suggests that improving feed efficiency via RFI selection does not negatively affect the set of quality parameters relevant to premium dry-cured ham production.

## Conclusions

This study suggests that sires with improved genetic merit for residual feed intake do not appear to negatively affect carcass traits or ham quality of their restricted-fed progeny. These findings provide preliminary evidence that genetic selection for feed efficiency based on RFI can be potentially compatible with the strict quality standards required for PDO dry-cured ham production. Importantly, these results are specific to RFI, which, unlike traditional measures such as feed conversion ratio, captures variation in feed intake independent of growth rate and tissue accretion, reflecting differences in maintenance and growth energy requirements and energy expenditure for physical activity. As such, RFI emerges as a suitable trait to include in breeding objectives for heavy pig sire lines. The results are based on a single population of heavy pigs reared under restricted feeding with a limited sample size. Further research is needed to confirm these results across diverse genetic backgrounds, production environments, and over multiple generations of selection. Genetic correlations between RFI and ham quality traits are currently unknown, and estimating them is essential to develop sustainable and effective feed efficiency-focused selection strategies in the context of high-quality pork production. Without such information, long-term selection could lead to unanticipated trade-offs between efficiency and product quality.

## Supporting information

S1 Table Ingredient composition and nutrient content of diets. Description of the diets for the early (≤ 120 kg body weight) and late (> 120 kg body weight) finisher period.
(DOCX)

S2 Table Estimation of cumulative ham weight loss curve parameters. Least squares means (± SE) of Chapman-Richards curve parameters across RFI sire classes.
(DOCX)
