# Supplementary material for: Genetic merit of sires for ad libitum residual feed intake has no adverse effects on carcass and ham quality traits of restricted-fed heavy pigs
Source: PLoS One. 2026 Mar 19;21(3):e0345035. doi: 10.1371/journal.pone.0345035 (PMC13001969; doi:10.1371/journal.pone.0345035)
Supplement: S1 Table — Description of the diets for the early (≤ 120 kg body weight) and late (> 120 kg body weight) finisher period. (DOCX) [file pone.0345035.s001.docx]

**S1 Table. Ingredient composition and nutrient content of diets.**

| **Diet Composition^b^** | **Feeding group^a^** | | | | | |
| --- | --- | --- | --- | --- | --- | --- |
|  | ***Ad libitum*** | | **Restricted** | | | |
|  |  |  | **MP** | | **LP** | |
|  | ≤ 120 kg BW | > 120 kg BW | ≤ 120 kg BW | > 120 kg BW | ≤ 120 kg BW | > 120 kg BW |
| DM, g/kg as fed | 906 | 906 | 904 | 902 | 904 | 904 |
| Ingredient Composition (% on DM) | | | | | | |
| Corn grain | 35.09 | 38.87 | 34.20 | 39.02 | 38.17 | 39.11 |
| Wheat grain | 24.95 | 24.84 | 28.20 | 24.89 | 27.24 | 24.93 |
| Barley grain | 9.64 | 9.69 | 9.70 | 9.73 | 9.72 | 9.74 |
| Soybean meal 48% (solv. ex.) | 20.10 | 14.73 | 8.70 | 5.79 | 3.93 | 1.89 |
| Wheat bran | 2.55 | 0.72 | 8.44 | 5.58 | 8.26 | 6.07 |
| Wheat middlings | - | 3.91 | 1.96 | 6.63 | 2.94 | 8.85 |
| Cane molasses | 1.60 | 1.81 | 1.61 | 1.82 | 1.63 | 1.82 |
| Lard | 2.21 | 2.22 | 2.37 | 2.23 | 2.34 | 2.23 |
| Dried-sugar beet pulp | - | - | 0.99 | 0.99 | 1.98 | 2.04 |
| Calcium carbonate | 1.66 | 1.44 | 1.66 | 1.45 | 1.67 | 1.45 |
| Dicalcium phosphate | 0.48 | 0.22 | 0.48 | 0.22 | 0.49 | 0.22 |
| Sodium chloride | 0.33 | 0.33 | 0.33 | 0.33 | 0.33 | 0.33 |
| Sodium bicarbonate | 0.27 | 0.28 | 0.28 | 0.28 | 0.28 | 0.28 |
| Vitamin and mineral premix | 0.20 | 0.20 | 0.20 | 0.20 | 0.20 | 0.20 |
| Grapeseed meal | 0.73 | 0.74 | 0.74 | 0.74 | 0.74 | 0.74 |
| Choline, liquid, 75% | 0.06 | - | - | - | - | - |
| L-Lysine | 0.11 | - | 0.15 | 0.11 | 0.07 | 0.11 |
| DL-Methionine | 0.02 | - | - | - | - | - |
| Nutrient Content | | | | | | |
| Metabolizable energy (MJ/kg DM) | 14.80 | 14.80 | 14.60 | 14.60 | 14.60 | 14.50 |
| Net energy (MJ/kg DM) | 11.00 | 11.10 | 11.10 | 11.10 | 11.20 | 11.00 |
| Crude protein (% on DM) | 17.88 | 15.23 | 14.16 | 13.19 | 12.50 | 11.5 |
| Lysine (% on DM) | 0.96 | 0.75 | 0.73 | 0.55 | 0.52 | 0.40 |
| Methionine (% on DM) | 0.30 | 0.28 | 0.24 | 0.22 | 0.21 | 0.20 |
| Threonine (% on DM) | 0.72 | 0.55 | 0.50 | 0.48 | 0.48 | 0.39 |
| Tryptophan (% on DM) | 0.20 | 0.14 | 0.17 | 0.12 | 0.13 | 0.11 |
| Tyrosine (% on DM) | 0.61 | 0.38 | 0.42 | 0.37 | 0.38 | 0.29 |
| Fatty acid profile | | | | | | |
| 16:0 (% on DM) | 8.06 | 8.10 | 8.85 | 8.54 | 8.85 | 8.63 |
| 18:0 (% on DM) | 3.09 | 3.09 | 3.32 | 3.10 | 3.21 | 3.10 |
| c18:1 (% on DM) | 13.47 | 13.58 | 14.38 | 13.97 | 14.38 | 13.94 |
| c18:2n-6 (% on DM) | 12.58 | 12.91 | 14.05 | 14.08 | 14.49 | 14.38 |
| c18:3n-3 (% on DM) | 0.77 | 0.77 | 0.88 | 0.89 | 0.88 | 0.88 |

Description of diet for the early (≤ 120 kg body weight, BW) and late (> 120 kg BW) finisher period.

^a^ MP: medium-protein diet; LP: low-protein diet.

^b^ Vitamin and mineral premix: providing per kilogram of feed; vitamin A, 8000 IU; vitamin D3, 1200 IU; vitamin E, 8 mg; vitamin B7, 0.08 mg; vitamin B12, 0.012 mg; niacin, 16.0 mg; biotin, 8 mg; iron, 170 mg; zinc, 117 mg; copper, 14 mg; cobalt, 0.11 mg; iodine, 0.06 mg; manganese, 65 mg; magnesium, 0.14 mg; selenium 10 mg; L-Lysine: Monochlorohydrate, 98.5% purity, 78% L-Lysine; DL-Methionine: 98% purity min.
