# Supplementary material for: Genetic merit of sires for ad libitum residual feed intake has no adverse effects on carcass and ham quality traits of restricted-fed heavy pigs
Source: PLoS One. 2026 Mar 19;21(3):e0345035. doi: 10.1371/journal.pone.0345035 (PMC13001969; doi:10.1371/journal.pone.0345035)
Supplement: S2 Table — Least squares means (± SE) of Chapman-Richards curve parameters across RFI sire classes. (DOCX) [file pone.0345035.s002.docx]

**S2 Table. Estimates of cumulative ham weight loss curve parameters.** Least square means (± SE) of Chapman-Richards curve parameters across sire RFI classes.

|  | **Sire classification** | | | **Contrast (*P-value*)** | |
| --- | --- | --- | --- | --- | --- |
| **Parameter^a^** | **High-RFI** | **Medium-RFI** | **Low-RFI** | **Low- vs Medium-RFI** | **Low- vs High-RFI** |
| n | 61 | 81 | 70 |  |  |
| *A* | 29.80 ± 0.68 | 29.90 ± 0.67 | 29.60 ± 0.68 | 0.345 | 0.537 |
| *k* | 0.0059 ± 0.00039 | 0.0058 ± 0.00039 | 0.0058 ± 0.00039 | 0.435 | 0.346 |
| *b* | 0.804 ± 0.032 | 0.792 ± 0.031 | 0.789 ± 0.031 | 0.157 | 0.104 |

^a^*A* (%) is the upper asymptotic CWL achievable for time → ∞; *k* (1/day) is the coefficient of loss affecting the steepness of the resulting curve, indicating how rapidly CWL approaches A; *b* is the curve shape parameter, controlling the curvature of the trajectory.
